# Relationship Between Measures of Cerebrovascular Reactivity and Intracranial Lesion Progression in Acute TBI Patients: an Exploratory Analysis

**DOI:** 10.1007/s12028-019-00885-3

**Published:** 2019-12-03

**Authors:** François Mathieu, Frederick A. Zeiler, Daniel P. Whitehouse, Tilak Das, Ari Ercole, Peter Smielewski, Peter J. Hutchinson, Marek Czosnyka, Virginia F. J. Newcombe, David K. Menon

**Affiliations:** 1 Division of Neurosurgery, University of Toronto, Toronto, Canada; 2 Division of Anaesthesia, Addenbrooke’s Hospital, University of Cambridge, Hills Road, Box 93, Cambridge, CB2 0QQ UK; 3 Section of Neurosurgery, Department of Surgery, Rady Faculty of Health Sciences, University of Manitoba, Winnipeg, Canada; 4 Department of Radiology, Addenbrooke’s Hospital, Cambridge University Hospital NHS Foundation Trust, Addenbrooke’s Hospital, Hills Road, Box 218, Cambridge, CB2 0QQ UK; 5 Brain Physics Laboratory, Division of Neurosurgery, Addenbrooke’s Hospital, Cambridge University Hospital NHS Foundation Trust, Cambridge, UK; 6 Brain Physics LaboratoryDivision of Neurosurgery, Addenbrooke’s Hospital, University of Cambridge, Hills Road, Box 167, Cambridge, CB2 0QQ UK; 7 Institute of Electronic Systems, Warsaw University of Technology, Warsaw, Poland; 8 Department of Clinical Neurosciences, University of Cambridge, Cambridge, UK; 9 Department of Anatomy and Cell Science, Rady Faculty of Health Sciences, University of Manitoba, Winnipeg, Canada; 10 Biomedical Engineering, Faculty of Engineering, University of Manitoba, Winnipeg, Canada

**Keywords:** Neurophysiological monitoring, Traumatic brain injury, Traumatic intracranial hemorrhage

## Abstract

**Background:**

Failure of cerebral autoregulation and progression of intracranial lesion have both been shown to contribute to poor outcome in patients with acute traumatic brain injury (TBI), but the interplay between the two phenomena has not been investigated. Preliminary evidence leads us to hypothesize that brain tissue adjacent to primary injury foci may be more vulnerable to large fluctuations in blood flow in the absence of intact autoregulatory mechanisms. The goal of this study was therefore to assess the influence of cerebrovascular reactivity measures on radiological lesion expansion in a cohort of patients with acute TBI.

**Methods:**

We conducted a retrospective cohort analysis on 50 TBI patients who had undergone high-frequency multimodal intracranial monitoring and for which at least two brain computed tomography (CT) scans had been performed in the acute phase of injury. We first performed univariate analyses on the full cohort to identify non-neurophysiological factors (i.e., initial lesion volume, timing of scan, coagulopathy) associated with traumatic lesion growth in this population. In a subset analysis of 23 patients who had intracranial recording data covering the period between the initial and repeat CT scan, we then correlated changes in serial volumetric lesion measurements with cerebrovascular reactivity metrics derived from the pressure reactivity index (PRx), pulse amplitude index (PAx), and RAC (correlation coefficient between the pulse amplitude of intracranial pressure and cerebral perfusion pressure). Using multivariate methods, these results were subsequently adjusted for the non-neurophysiological confounders identified in the univariate analyses.

**Results:**

We observed significant positive linear associations between the degree of cerebrovascular reactivity impairment and progression of pericontusional edema. The strongest correlations were observed between edema progression and the following indices of cerebrovascular reactivity between sequential scans: % time PRx > 0.25 (*r *= 0.69, *p* = 0.002) and % time PAx > 0.25 (*r *= 0.64, *p* = 0.006). These associations remained significant after adjusting for initial lesion volume and mean cerebral perfusion pressure. In contrast, progression of the hemorrhagic core and extra-axial hemorrhage volume did not appear to be strongly influenced by autoregulatory status.

**Conclusions:**

Our preliminary findings suggest a possible link between autoregulatory failure and traumatic edema progression, which warrants re-evaluation in larger-scale prospective studies.

## Introduction

Impairment of cerebrovascular autoregulatory mechanisms has been shown to be associated with worse global outcome in neurologically injured patients, particularly in the acute traumatic brain injury (TBI) population [2, 3]. The most commonly used continuous indices of global cerebral autoregulation are based on moving correlation coefficients between mean arterial (MAP) or cerebral perfusion pressure (CPP) and a surrogate of cerebral blood volume, typically inferred from continuous measurements of intracranial pressure (ICP). The pressure reactivity index (PRx), for example, is derived from the correlation between ICP and MAP and has shown strong independent association with mortality and morbidity at 6 months in a retrospective cohort of TBI patients [4]. Indices based on the pulse amplitude of ICP (AMP) which better reflect compliance such as PAx (the moving correlation between MAP and AMP) and RAC (the moving correlation between CPP and AMP) have also been linked to outcome and may be more discriminating than PRx at lower thresholds of ICP [5, 6]. However, the pathophysiological mechanisms through which impaired cerebrovascular reactivity contributes to poorer outcome remain unclear.

One candidate for translation of such abnormal pathophysiology to structural injury may be lesion progression. Delayed lesion expansion can lead to worsened secondary injury and has also been associated with poorer outcome [7–10]. Patients with radiological progression are also more likely to require surgical procedures, thereby putting them at risk of surgery-related complications and contributing to the length of stay and overall treatment and rehabilitation costs [9, 11].

Preliminary evidence from animal studies indicates that the energy transferred at the time of injury affects cerebrovascular responses in both the hemorrhagic lesion core and perilesional zones and can impair microvascular function even in the absence of overt structural vascular damage [12, 13]. We hypothesized that impaired autoregulation could contribute to traumatic lesion expansion by decreasing the brain’s capacity to protect itself from fluctuations in arterial pressures, thus subjecting vulnerable downstream microvessels to high stress. This issue has not, to our knowledge, been formally investigated in the past. However, documenting such a relationship could improve precision medicine approaches to TBI management, by identifying a risk factor for lesion expansion, establishing a proximate structural consequence of physiological derangements, informing the frequency and timing of follow up imaging, and stratifying patients at high risk of needing surgical intervention and more aggressive medical therapies.

The goal of this study was therefore to explore the relationship between indices of cerebrovascular reactivity and traumatic lesion progression in a cohort of acute TBI patients. Given the wide range of autoregulatory indices available, and the range of summary measures that can be derived for each of these, we also wished to determine which of these showed the strongest associations with hemorrhagic or edema expansion, so as to provide the best candidates for a subsequent confirmatory study.

## Methods

This was a retrospective analysis of a prospectively maintained database cohort, in which high-frequency clinical neuromonitoring data had been archived. Monitoring of brain modalities was conducted between October 2006 and April 2014 as a part of standard neurosciences critical care unit (NCCU, Addenbrooke’s Hospital Cambridge) patient care. Relevant clinical data were recorded at the time of monitoring, and no attempt was made to re-access clinical records for additional information. Since all data were extracted from routine hospital records and fully anonymized before analysis, the need for formal review and consent not required. Such research use of routine clinical data, originally collected as part of ongoing clinical care, without the intention of using it for research at the time of collection, complies with UK Governance Arrangements for Research Ethics Committees (GAfREC), 2018 [1], and this was reconfirmed through electronic communication with the UK National Health Service Health Research Authority (document on file).

### Study Population

A total of 50 patients were included in this study. Inclusion criteria were age > 18 years, a diagnosis of TBI requiring admission to the neurointensive care unit for sedation and ventilation, the presence of archived high-frequency digital physiology from multimodality monitoring performed as part of clinical care, availability of an admission computed tomography (CT) scan and repeat scan performed during the acute period, defined within 7 days of injury. Patients who underwent a decompressive craniectomy were excluded unless two CT scans had been performed prior to surgical decompression. This decision was based on the difficulties in making reliable comparisons between serial lesion measurements in this subgroup and the impact of craniectomy on measurement of cerebrovascular reactivity indices [36]. Patients were managed according to the TBI treatment guidelines in effect during the study period, including ICP-directed therapy aimed at maintaining an ICP of less than 20 mm Hg and a CPP of greater than 60 mm Hg [14].

## Data Collection

Baseline demographic (age, gender) and clinical data (Glasgow Coma Scale (GCS) score, pupillary reactivity) were recorded at the time of admission to our facility (admission GCS was scored pre-intubation). Data collected included the length of stay in the NCCU and 6-month functional outcome when available (Glasgow Outcome Scale).

### Intracranial Monitoring

Arterial blood pressure was measured through a radial or femoral line connected to a pressure transducer (Baxter Healthcare Corp, CardioVascular Group, Irvine, CA, USA). When the clinical team determined that invasive intracranial neuromonitoring was indicated, an intra-parenchymal ICP monitor (Codman ICP MicroSensor, Codman & Shurtleff Inc., Raynham, MA, USA) was placed using triple bolt intracranial access kit [15]. Signals were digitized using an A/D converter (DT9800 series, Data Translation, Marlboro, MA, USA) and sampled using ICM + software (Cambridge Enterprise Ltd, Cambridge, UK, http://www.neurosurg.cam.ac.uk/icmplus).

Processing of the acquired signals was performed using ICM + software as previously described [5, 6]. The focus was on the relationship between slow waves of ICP, arterial blood pressure and CPP (CPP = MAP–ICP), as such the parent high-frequency signals were downsampled to 0.1 Hz using a 10-s moving average filter. AMP was defined as the Fourier amplitude of the ICP pulse waveform over this window, updated every 10 s. PRx, PAx, and RAC were derived by calculating moving correlation coefficients between slow-wave fluctuations ICP and MAP, AMP and MAP, or AMP and CPP, respectively, using 30 consecutive 10-s windows updated every minute. For all of these measures, more positive measurement values represent greater impairment in cerebrovascular reactivity (PRx, PAx) or combined cerebrovascular reactivity and cerebrovascular compensatory reserve (RAC) [6, 16].

### Imaging

**Fig. 1:**
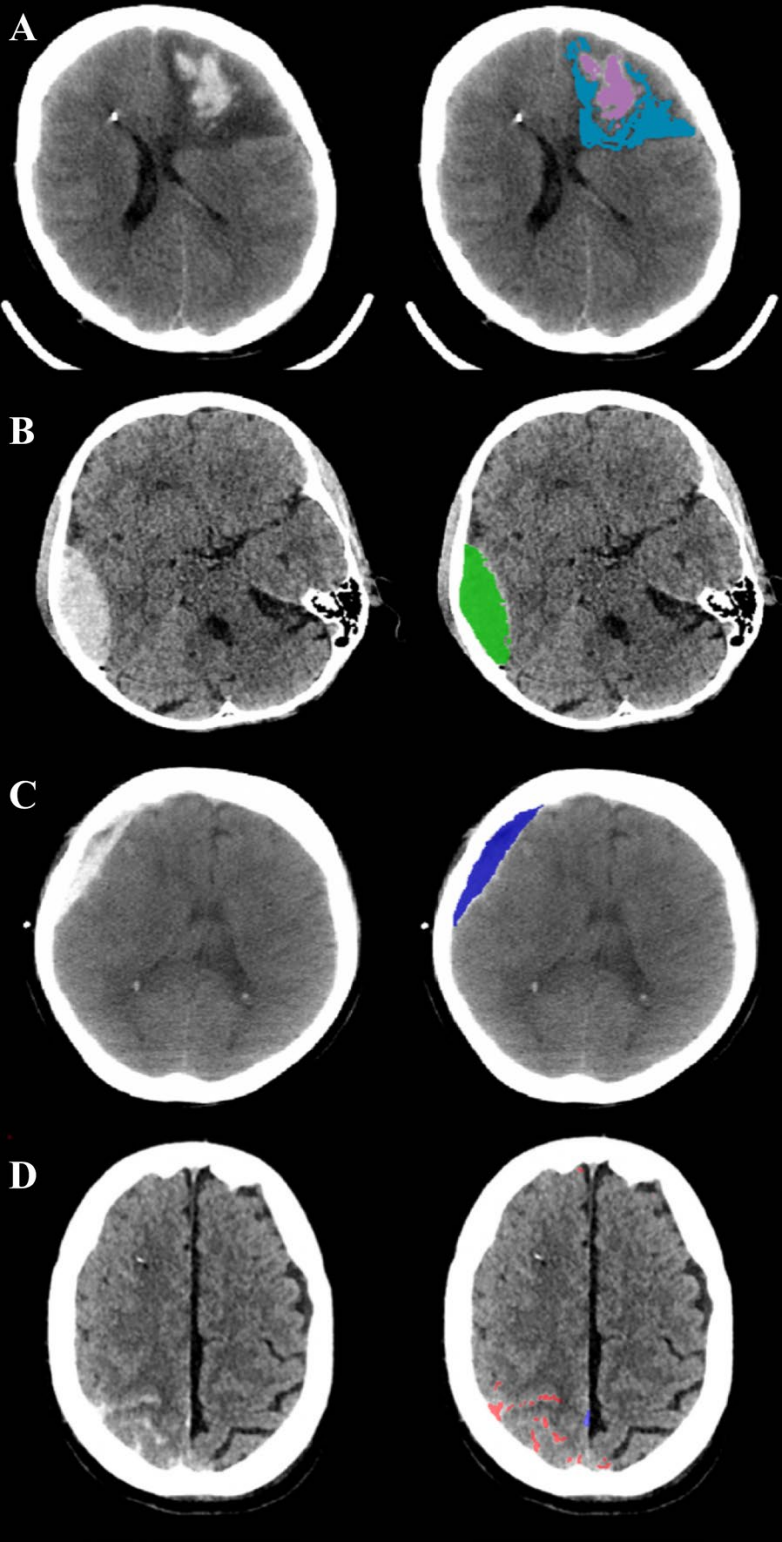
Representative set of segmented traumatic lesions on CT. Left: initial image; Right: segmented lesion(s). **a** Contusion core (blue) and peri-contusional edema (magenta), **b** epidural hematoma (green), **c** subdural hematoma (dark blue), **d** subarachnoid hemorrhage (red) and trace parafalcine extra-axial blood (dark blue). Right frontal hyperdensity in **a** and **d** represents the tip of the intracranial probe

Axial CT scans for each patient were downloaded in a Digital Imaging and Communication in Medicine format, assigned a study identifier that associated it with the anonymized physiological and clinical data available for the patient, and converted to anonymized Neuroimaging Informatics Technology Initiative format for analysis. Traumatic intracranial lesions were digitally segmented by an expert clinician, a method which has shown superior accuracy to the commonly used pragmatic volume measurements such as ellipsoid or Cavalieri approximation [17]. A bespoke image segmentation tool developed in the BioMedIA group at Imperial College London with a set of labels customized for traumatic brain lesion annotations was used (ImSeg, v1.9, Imperial College London, UK). This technique has demonstrated adequate inter- and intra-observer reliability for our imaging dataset, with inter-rater intraclass correlation coefficients (ICCs) of 0.97 (95% CI 0.92–0.99), 0.97 (95% CI 0.90–0.99), and .98(95% CI .94–.99) for contusion core, edema and extra-axial hemorrhage, respectively, and intra-rater ICCs of 0.98 (95% CI 0.92–0.99), 0.97 (95% CI 0.90–0.99), and 0.98(95% CI 0.94–0.99), respectively. These reliability coefficients are in line with those reported in a larger study of intracerebral hemorrhage measurements on CT [18]. Extra-axial lesions were divided into epidural, subdural, and subarachnoid hemorrhage. Intra-parenchymal lesions were parcellated based on type (core versus edema, nontraumatic). A representative set of segmentations is shown in Fig. 1. Segmentations were validated by a second clinician with specialist expertise in neuroradiology. The clinicians assessing the lesions were blinded to the patient’s cerebrovascular reactivity status. Segmented lesion volumes were extracted using FSL v6.0 (NeuroImage, https://fsl.fmrib.ox.ac.uk/fsl/fslwiki/FSL) for subsequent analyses.

### Outcome Measures

The exposure of interest was autoregulatory status in the acute phase of TBI, and the primary outcome was radiological lesion progression during this same period. Lesion progression was calculated by subtracting absolute lesion volumes (measured in mL) on the initial scan from the repeat scan. Total averages [19] and percent of monitoring time spent above thresholds were calculated for each cerebrovascular reactivity index for the interval between the initial and repeat scan. The critical thresholds of 0.25 for PRx and PAx and − 0.10 for RAC were chosen based on the previous literature suggesting that these cutoffs predict poor 6-month functional outcome in non-craniectomized TBI patients [20].

### Statistical Analysis

Data were analyzed using R (R Core Team (2016); R: a language and environment for statistical computing; R Foundation for Statistical Computing, Vienna, Austria; URL https://www.R-project.org/) and JMP Pro software (version 14, SAS Institute Inc., Cary, NC, 1989–2019). For all reported *p* values, the alpha was set as 0.05 for significance.

The descriptive statistics used to assess the characteristics of our cohort are reported as mean (standard deviation) for normally distributed continuous variable and median (range or interquartile range) for ordinal data, and categorical variables are represented as proportions of the total sample. Generalized linear regression was used to look at associations between non-neurophysiological factors including initial lesion size, interval between the initial and repeat scan, the presence of a documented coagulopathy during this interval (defined as a PT > 13.5 s, aPTT > 35 s or platelets < 100/nL), and traumatic lesion growth. We also included mean CPP as another potential confounding factor, based on the concern that a relatively higher CPP target in patients with more severe intracranial injuries could contribute to hemorrhage or edema expansion [21, 22]. Scatter plot matrices, Pearson correlation analyses, and linear mixed-model regression were then used to look for linear relationships between cerebrovascular reactivity metrics and lesion change in the subset of patients with intracranial recordings covering the between-scan interval. Correlation and regression coefficients were calculated for each lesion label as well as collapsed categories (total contusion core, total contusion-related edema, and total extra-axial hemorrhage volume). Using multivariate generalized linear mixed regression methods, these coefficients were adjusted for non-neurophysiological parameters associated with lesion growth and CPP based on variables with a *p* value < 0.2 from the univariate analysis, as well as baseline covariate including age, gender, and GCS on admission. Significant *p* values were tested against thresholds corrected for multiple comparisons using the Holm–Bonferroni method for both the adjusted and unadjusted models.

## Results

### Patient Characteristics

The patient demographics and clinical characteristics for the entire cohort (*n *= 50) are summarized in Table 1. Admission (initial) CT scans were obtained within 7.3 ± 10.4 h of injury. Initial imaging findings and mean neurophysiological values over the recording period are presented in Table 2. Given that the available repeat imaging studies were obtained within a mean of 62.4 ± 64.8 h of injury and that there were delays to initiation of monitoring for many patients (mean delay = 45.6 ± 31.2 h), only 23 of those patients had physiological recordings data available for the period between the initial and repeat scans.

**Table 1:** Patient demographics and baseline clinical characteristics

Characteristics	Mean/median (SD or range)
Number of patients	50
Age, years	35.6 (15.6)
Female, *n* (%)	15 (30)
Admission GCS total	6 (1–15)
Admission GCS motor	3 (1–6)
Pupils, *n* (%)	
Unilateral sluggish	2 (4)
Bilateral sluggish	3 (6)
Unilateral unreactive	4 (8)
Bilateral unreactive	6 (12)
Length of stay in ICU, *mean days*	23 (8.8)
GOS at 6 months, *n* (%)	
1	4 (8)
2	19 (38)
3	16 (32)
4	7 (14)
Unavailable	4 (8)

*GCS* Glasgow Coma Scale, *GOS* Glasgow Outcome Scale, *SD* standard deviation

**Table 2:** Initial imaging findings and neurophysiological characteristics

	Mean/median (SD or range)
Initial CT scan	
Marshall score	2 (1–6)
Rotterdam score	3 (1–6)
ASDH, *n* (%)	24 (48)
EDH, *n* (%)	7 (14)
SAH, *n* (%)	26 (52)
IVH, *n* (%)	15 (30)
Contusion, *n* (%)	41 (82)
Superficial	39 (78)
Deep	16 (32)
Contusion volume, *n* (%)	
< 10 mL	36 (72)
10–25 mL	2 (4)
> 25 mL	3 (6)
Timing of initial scan, *mean hours from injury*	7.3 (10.4)
Timing of repeat scan, *mean hours from injury*	62.4 (64.8)
Length of physiological recordings, *mean days*	7.4 (5.4)
Mean ICP, mmHg	15.3 (9.7)
Mean CPP, mmHg	80.3 (7.9)
Mean PRx, *a.u.*	0.03 (0.14)
Mean PAx, *a.u.*	−0.09 (0.16)
Mean RAC, *a.u.*	−0.39 (0.22)

*n *= number of patients with the type of lesion on initial CT (% of total sample); *a.u*. arbitrary units, *ASDH* acute subdural hematoma, *CPP* cerebral perfusion pressure, *EDH* epidural hematoma, *ICP* intracranial pressure, *IVH* intraventricular hemorrhage, *PAx* pulse amplitude index (correlation between pulse amplitude of intracranial pressure and mean arterial pressure), *PRx* pressure reactivity index (correlation between intracranial pressure and mean arterial pressure), *RAC* correlation between pulse amplitude of intracranial pressure and cerebral perfusion pressure, *SAH* subarachnoid hemorrhage, *SD* standard deviation

### Association Between Non-neurophysiological Factors and Absolute Lesion Change

Univariate regression analyses performed on the full cohort (*n *= 50) showed positive linear relationships between initial lesion volume, mean CPP, and progression of pericontusional edema (Table 3). There were no significant relationships between initial lesion volume, number of hours elapsed between initial and repeat scan, mean CPP, and the presence of a coagulopathy in the between-scan interval and growth of hemorrhagic core or extra-axial hemorrhage volume. *P* values for the associations between initial lesion volume, mean CPP, time elapsed between scans, and growth of lesion core were < 0.20 and therefore entered in the multivariate models.

**Table 3:** Univariate associations for factors previously associated with lesion growth (*n *= 50)

	Δ Core			Δ Edema			Δ Extra-axial		
	Coefficient (95% CI)	*R*^2^	*p* value	Coefficient (95% CI)	*R*^2^	*p* value	Coefficient (95% CI)	*R*^2^	*p* value
Initial lesion volume (mL)	0.16 [− 0.02 to 0.34]	0.08	0.07	0.64 [0.38 to 0.90]	0.42	< 0.0001	0.03 [− 0.13 to 0.21]	0	0.76
Interval between scans	− 0.01 [− 0.03 to 0.01]	0.04	0.19	− 0.001 [− 0.04 to 0.04]	0	0.69	− 0.01 [− 0.03 to 0.01]	0.06	0.20
Mean CPP	0.14 [− 0.04 to 0.32]	0.06	0.14	0.37 [0.01 to 0.73]	0.1	0.05	− 0.01 [− 0.19 to 0.17]	0	0.89
Coagulopathy	0.60 [− 1.0 to 2.19]	0.02	0.44	0.28 [− 2.95 to 3.51]	0	0.86	0.26 [− 1.08 to 1.60]	0.04	0.70

Δ Core, absolute difference in volume (mL) for contusion core between initial and repeat scan; Δ Edema, absolute difference in volume (mL) for pericontusional edema between initial and repeat scan; Δ Extra-axial, absolute difference in total volume (mL) of extra-axial hemorrhage between initial and repeat scan, *CPP* cerebral perfusion pressure

### Association Between Cerebrovascular Reactivity Measurements and Absolute Lesion Volume Change

**Fig. 2:**
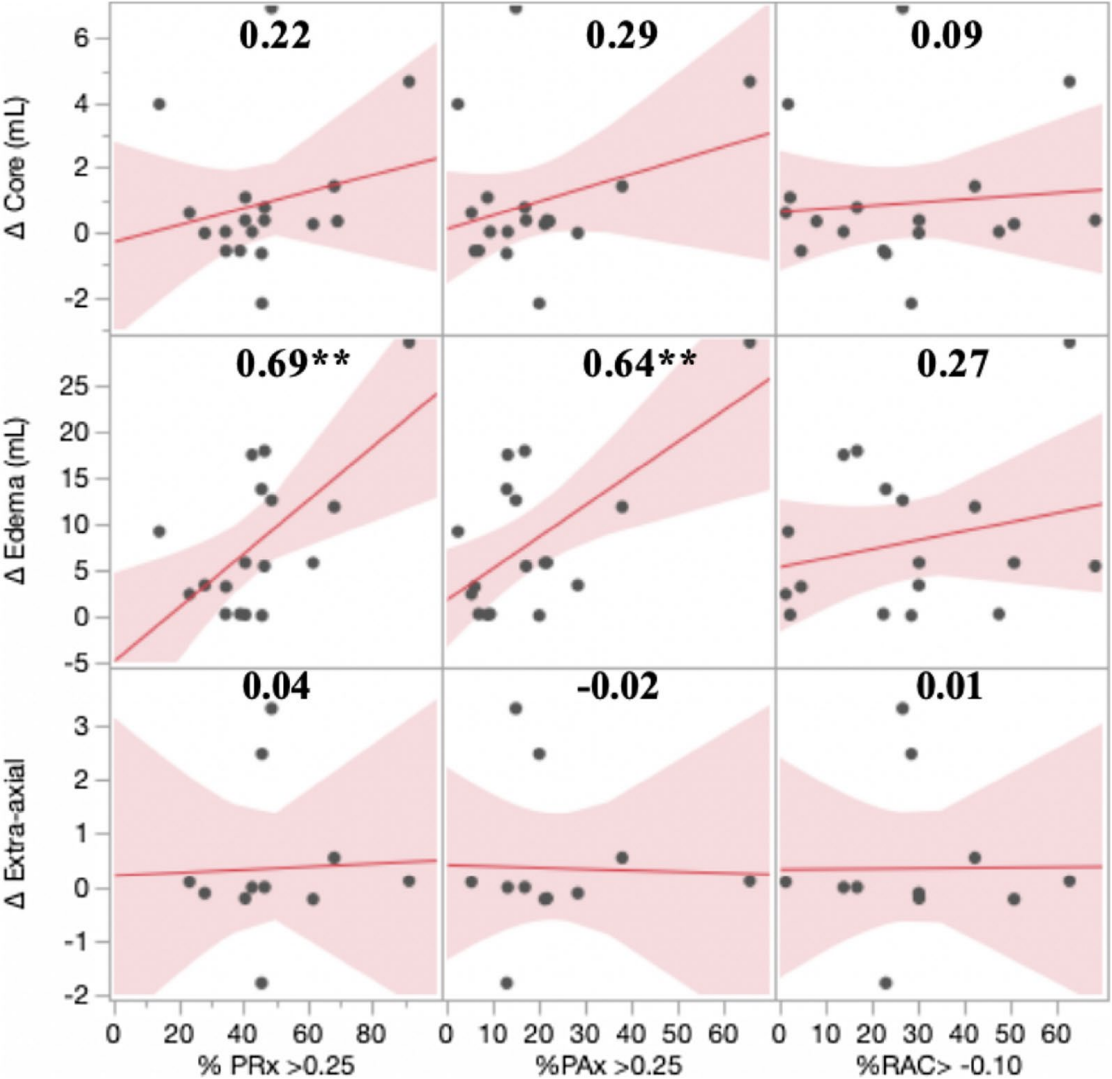
Scatter plot matrix of correlations between cerebrovascular reactivity status and radiological lesion progression. Recording interval between initial and repeat scan (*n *= 23). Correlation coefficients are shown in the top middle portion of each scatter plot. Δ Core, absolute difference in volume (mL) for contusion core between initial and repeat scan; Δ Edema, absolute difference in volume (mL) for peri-contusional edema between initial and repeat scan; Δ Extra-axial, absolute difference in total volume (mL) of extra-axial hemorrhage between initial and repeat scan, *PAx* pulse amplitude index (correlation between pulse amplitude of intracranial pressure and mean arterial pressure), *PRx* pressure reactivity index (correlation between intracranial pressure and mean arterial pressure, *RAC* correlation between pulse amplitude of intracranial pressure and cerebral perfusion pressure

Pearson correlational analyses showed significant positive linear associations between the degree of cerebrovascular reactivity impairment and progression of pericontusional edema. Scatter plots with corresponding correlation coefficients for relevant pairs of variables are displayed in Fig. 2. The strongest associations were observed between edema progression and percent time spent above a threshold of PRx > 0.25 (*r *= 0.69, *p *= 0.002) or PAx > 0.25 (*r *= 0.64, *p *= 0.006). In contrast, we found no significant correlations between any of the cerebrovascular reactivity metrics and progression of contusion core or extra-axial hemorrhage. We noted that an outlier appeared to be influencing correlations in both directions depending on the variable pair (Fig. 2). In a sensitivity analysis, a test exclusion of this data point did not change the overall significance levels for the majority of associations presented. The associations between the degree of cerebrovascular reactivity impairment and edema expansion held true after adjustment for baseline covariates, potential non-neurophysiological factors, and mean CPP (Tables 4, 5) and after correcting for multiple comparisons. We decided to exclude extra-axial hemorrhage from the multivariate models given the lack of associations seen in the univariate analyses.

**Table 4:** Linear associations between cerebrovascular reactivity impairment and contusion core progression (*n *= 23)

	Δ Core (unadjusted)			Δ Core (adjusted)		
	Coefficient (95% CI)	*R*^2^	*p* value	Coefficient (95% CI)	*R*^2^ (*R*^2^-adj)	*p* value
% Time spent PRx > 0.25	0.02 [− 0.02 to 0.07]	0.05	0.37	− 0.05 [− 0.17 to 0.05 ]	0.43 (0.13)	0.29
% Time spent PAx > 0.25	0.04 [− 0.02 to 0.11]	0.08	0.25	− 0.05 [− 0.24 to 0.13)	0.39 (0.06)	0.53
% Time spent RAC > − 0.10	0.01 [− 0.04 to 0.14]	0.01	0.71	− 0.03 [− 0.10 to 0.05]	0.41 (0.09)	0.41

Δ Core, absolute difference in volume (mL) for contusion core between initial and repeat scan; %, percent time spent above respective thresholds; *PAx* pulse amplitude index (correlation between pulse amplitude of intracranial pressure and mean arterial pressure); *PRx* pressure reactivity index (correlation between intracranial pressure and mean arterial pressure);%, percent time spent above respective thresholds; *Hrly* hourly dose above respective thresholds, *RAC* correlation between pulse amplitude of intracranial pressure and cerebral perfusion pressure

**Table 5:** Linear associations between cerebrovascular reactivity impairment and pericontusional edema progression (*n *= 23)

	Δ Edema (unadjusted)			Δ Edema (adjusted)		
	Coefficient (95% CI)	*R*^2^	*p* value	Coefficient (95% CI)	*R*^2^ (*R*^2^-adj)	*p* value
% Time spent PRx > 0.25	0.30 [0.14 to 0.45 ]	0.47	< 0.01	0.36 [0.11 to 0.60]	0.58 (0.39)	< 0.01
% Time spent PAx > 0.25	0.34 [0.14 to 0.54]	0.41	< 0.01	0.62 [0.24 to 1.00]	0.63 (0.45)	< 0.01
% Time spent RAC > − 0.10	0.10 [− 0.09 to 0.18]	0.06	0.33	0.05 [− 0.19 to 0.30]	0.62 (0.40)	0.64

Δ Edema, absolute difference in volume (mL) for pericontusional edema between initial and repeat scan; %, percent time spent above respective thresholds, *PAx* pulse amplitude index (correlation between pulse amplitude of intracranial pressure and mean arterial pressure), *PRx* pressure reactivity index (correlation between intracranial pressure and mean arterial pressure);%, percent time spent above respective thresholds; Hrly, hourly dose above respective thresholds; *RAC* correlation between pulse amplitude of intracranial pressure and cerebral perfusion pressure. Unadjusted model: exact *p* values for % time spent with PRx or PAx > 0.25 were 0.0023 and 0.0058, respectively, tested against corrected thresholds of 0.05/9 = 0.0055 and 0.05/8 = 0.0063; Adjusted model: exact *p* values for % time spent with PRx or PAx > 0.25 were 0.0056 and 0.0042, respectively, testing against corrected threshold of 0.05/8 = 0.0063 and 0.05/9 = 0.0055.)

## Discussion

We sought to explore the relationship between cerebrovascular reactivity and traumatic intracranial lesion progression in a cohort of patients with acute traumatic brain injury and identify metrics of cerebrovascular reactivity that showed the strongest associations with lesion progression. Overall, our results suggest a link between autoregulatory dysfunction and expansion of perilesional edema in the acute phase, replicated across two metrics of autoregulatory dysfunction.

Prior studies have identified a number of factors contributing to contusion expansion in the traumatic setting, including age, initial contusion size, degree of overall mass effect, presence of coagulopathies, decompressive craniectomy, and timing of radiological assessment [7, 8, 10, 11, 23–33]. Few human studies, however, have attempted to isolate factors contributing to perilesional edema expansion separately from the hemorrhagic core. Preliminary evidence from animal data suggests that the kinetic energy transferred during the initial impact induces a molecular cascade which can trigger progressive microvascular failure in brain tissue adjacent to the primary injury site in the absence of overt vessel fracture [12, 13]. If this is the case, it is possible that loss of autoregulation may contribute to edema formation by allowing the transmission of elevated pressures to the grossly structurally intact, but functionally disrupted downstream microvasculature. A plausible alternative hypothesis would be that a larger edema volume is associated with greater impairment in cerebrovascular reactivity since it tends to reflect a more severe injury mechanism. However, this hypothesis is not supported by data showing a lack of significant association between total intracranial injury burden on admission CT scan and subsequent dysfunction in cerebrovascular reactivity [34]. While we cannot ascertain a direction of causality given the retrospective and highly exploratory nature of this study, this alternative hypothesis is also not supported by the lack of relationship between RAC-based measures and lesion expansion in our study. RAC is believed to incorporate additional information pertaining to cerebral compensatory reserve—the degree to which incremental changes in intracranial contents such as hemorrhagic or edematous lesions affect intracranial pressure—since it is derived from measuring changes in pulse amplitude of ICP in response to fluctuations in cerebral perfusion pressure [6, 35]. If dysfunction of cerebrovascular reactivity was primarily a by-product of increased mass effect from edema progression rather than a contributing cause, we would therefore expect this to be reflected by significant changes in the RAC index. In our study, the strongest associations with absolute lesion volume increase were in fact seen with PRx-based metrics, which has been the most widely studied thus far [36]. As the moving correlation between ICP and MAP, PRx is believed to primarily reflect the degree of intracerebral vessel reactivity [16]. Consequently, our results suggest that reactivity-dependent mechanisms may be involved in early traumatic edema development. However, we need to emphasize that our results are preliminary and that much more work will, however, be required to elucidate the temporal relationships between radiological lesion evolution and changes in the various surrogate measures of cerebrovascular function employed in this study.

It is unclear why extra-axial hemorrhage growth appeared to poorly correlate with autoregulatory status, but a number of potential contributing factors deserve mention. First, it is important to keep in mind that most of the larger extra-axial lesions or those deemed at risk of progression would have been surgically evacuated shortly after presentation. Such lesions would therefore have been excluded from our study, introducing a selection bias into the extra-axial analyses. Second, the source vascular supply for extra-axial bleeding—typically meningeal feeders supplied by branches of the extracranial circulation, dural venous sinuses or bone for epidural hematoma, and bridging veins for subdural hematoma—should in theory not be directly susceptible to cerebral autoregulatory dysfunction, although a subset of subdural hematomas is known to arise from extension of parenchymal contusion or cortical laceration across the pia-arachnoid membranes [37]. Third, conservatively managed extra-axial hemorrhages have been shown to redistribute over time, especially in older individuals with more pronounced brain atrophy, which may reduce the accuracy of serial volume measurements for this lesion subtype [38].

## Limitations

The results of this study should be interpreted bearing in mind a number of important limitations. First, the small sample size limited our ability to detect significant associations, especially when looking at less common lesion subtypes, and makes our analyses more vulnerable to outliers. Although we had originally planned to assess the influence of lesion location and subtype, this was not possible given the reduced sample size. Second, the retrospective nature of this study and the heterogeneity of our sample in terms of timing of radiological assessment and onset of monitoring make our analyses vulnerable to a range of selection and temporally dependent biases. Although a number of studies have shown that traumatic lesion expansion generally happens within the first 2 days of injury (mean repeat scan was obtained at 2.6 days in our study) [11, 31], patients with more severe injury may have been more likely to get an early repeat imaging study, confounding the assessment of a dynamic relationship with the intracranial recording data. Third, a minority of patients had femoral rather than radial lines and assessment of mean arterial pressures may therefore not be perfectly comparable among all subjects. Fourth, we cannot account for the influence of ICP-lowering therapies and vasoactive agents, which are commonly used in this patient population, as such interventions were not measured as part of this study and evidence regarding their impact on cerebrovascular responses is lacking. For instance, the clinical team may have targeted a higher CPP for more injured patients with positive indices, which could contribute to lesion progression. We should also point out that the continuous indices of cerebrovascular reactivity used in this study (PRx, PAx, RAC) reflect a global average of autoregulatory status and may not necessarily capture more focal physiological derangements that could influence lesion expansion.

## Conclusion

Despite these limitations, our findings point to a possible association between global autoregulatory failure and traumatic edema progression, which warrants re-evaluation in larger-scale prospective studies. Future studies should also incorporate functional outcome measures to determine how these lesion–autoregulation relationships influence patient recovery at various timepoints after the acute phase.

## References

[1] Governance arrangements for research ethics committees: a harmonised edition. . UK Health Departments; 2011.

[2] Rivera-Lara L, Zorrilla-Vaca A, Geocadin R, Ziai W, Healy R, Thompson R (2017). predictors of outcome with cerebral autoregulation monitoring: a systematic review and meta-analysis. Crit Care Med.

[3] Czosnyka M, Balestreri M, Steiner L, Smielewski P, Hutchinson PJ, Matta B (2005). Age, intracranial pressure, autoregulation, and outcome after brain trauma. J Neurosurg.

[4] Sorrentino E, Diedler J, Kasprowicz M, Budohoski KP, Haubrich C, Smielewski P (2012). Critical thresholds for cerebrovascular reactivity after traumatic brain injury. Neurocrit Care.

[5] Aries MJ, Czosnyka M, Budohoski KP, Kolias AG, Radolovich DK, Lavinio A (2012). Continuous monitoring of cerebrovascular reactivity using pulse waveform of intracranial pressure. Neurocrit Care.

[6] Zeiler FA, Donnelly J, Menon DK, Smielewski P, Hutchinson PJA, Czosnyka M (2018). A description of a new continuous physiological index in traumatic brain injury using the correlation between pulse amplitude of intracranial pressure and cerebral perfusion pressure. J Neurotrauma.

[7] Fainardi E, Chieregato A, Antonelli V, Fagioli L, Servadei F (2004). Time course of CT evolution in traumatic subarachnoid haemorrhage: a study of 141 patients. Acta Neurochir (Wien).

[8] Servadei F, Nanni A, Nasi MT, Zappi D, Vergoni G, Giuliani G (1995). Evolving brain lesions in the first 12 hours after head injury: analysis of 37 comatose patients. Neurosurgery.

[9] Stein SC, Spettell C, Young G, Ross SE (1993). Delayed and progressive brain injury in closed-head trauma: radiological demonstration. Neurosurgery.

[10] Chieregato A, Fainardi E, Morselli-Labate AM, Antonelli V, Compagnone C, Targa L (2005). Factors associated with neurological outcome and lesion progression in traumatic subarachnoid hemorrhage patients. Neurosurgery.

[11] Alahmadi H, Vachhrajani S, Cusimano MD (2010). The natural history of brain contusion: an analysis of radiological and clinical progression. J Neurosurg.

[12] Kurland D, Hong C, Aarabi B, Gerzanich V, Simard JM (2012). Hemorrhagic progression of a contusion after traumatic brain injury: a review. J Neurotrauma.

[13] Simard JM, Kilbourne M, Tsymbalyuk O, Tosun C, Caridi J, Ivanova S (2009). Key role of sulfonylurea receptor 1 in progressive secondary hemorrhage after brain contusion. J Neurotrauma.

[14] Bratton SL, Chestnut RM, Ghajar J, McConnell Hammond FF, Harris OA, Hartl R (2007). Guidelines for the management of severe traumatic brain injury. J Neurotrauma.

[15] Hutchinson PJ, Hutchinson DB, Barr RH, Burgess F, Kirkpatrick PJ, Pickard JD (2000). A new cranial access device for cerebral monitoring. Br J Neurosurg.

[16] Czosnyka M, Smielewski P, Kirkpatrick P, Laing RJ, Menon D, Pickard JD (1997). Continuous assessment of the cerebral vasomotor reactivity in head injury. Neurosurgery.

[17] Stocchetti N, Croci M, Spagnoli D, Gilardoni F, Resta F, Colombo A (2000). Mass volume measurement in severe head injury: accuracy and feasibility of two pragmatic methods. J Neurol Neurosurg Psychiatry.

[18] Zimmerman RD, Maldjian JA, Brun NC, Horvath B, Skolnick BE (2006). Radiologic estimation of hematoma volume in intracerebral hemorrhage trial by CT scan. AJNR Am J Neuroradiol.

[19] Zeiler FA, Donnelly J, Smielewski P, Menon DK, Hutchinson PJ, Czosnyka M (2018). Critical thresholds of intracranial pressure-derived continuous cerebrovascular. J Neurotrauma.

[20] Zeiler FA, Donnelly J, Smielewski P, Menon DK, Hutchinson PJ, Czosnyka M (2018). Critical thresholds of intracranial pressure-derived continuous cerebrovascular reactivity indices for outcome prediction in noncraniectomized patients with traumatic brain injury. J Neurotrauma.

[21] White CL, Griffith S, Caron JL (2009). Early progression of traumatic cerebral contusions: characterization and risk factors. J Trauma.

[22] Beaumont A, Hayasaki K, Marmarou A, Barzo P, Fatouros P, Corwin F (2000). The effects of dopamine on edema formation in two models of traumatic brain injury. Acta Neurochir Suppl.

[23] Gudeman SK, Kishore PR, Miller JD, Girevendulis AK, Lipper MH, Becker DP (1979). The genesis and significance of delayed traumatic intracerebral hematoma. Neurosurgery.

[24] Stein SC, Young GS, Talucci RC, Greenbaum BH, Ross SE (1992). Delayed brain injury after head trauma: significance of coagulopathy. Neurosurgery.

[25] Tseng SH (1992). Delayed traumatic intracerebral hemorrhage: a study of prognostic factors. J Formos Med Assoc.

[26] Patel NY, Hoyt DB, Nakaji P, Marshall L, Holbrook T, Coimbra R (2000). Traumatic brain injury: patterns of failure of nonoperative management. J Trauma.

[27] Oertel M, Kelly DF, McArthur D, Boscardin WJ, Glenn TC, Lee JH (2002). Progressive hemorrhage after head trauma: predictors and consequences of the evolving injury. J Neurosurg.

[28] Engstrom M, Romner B, Schalen W, Reinstrup P (2005). Thrombocytopenia predicts progressive hemorrhage after head trauma. J Neurotrauma.

[29] Chang EF, Meeker M, Holland MC (2007). Acute traumatic intraparenchymal hemorrhage: risk factors for progression in the early post-injury period. Neurosurgery.

[30] Flint AC, Manley GT, Gean AD, Hemphill JC, Rosenthal G (2008). Post-operative expansion of hemorrhagic contusions after unilateral decompressive hemicraniectomy in severe traumatic brain injury. J Neurotrauma.

[31] Narayan RK, Maas AI, Servadei F, Skolnick BE, Tillinger MN, Marshall LF (2008). Progression of traumatic intracerebral hemorrhage: a prospective observational study. J Neurotrauma.

[32] Allard CB, Scarpelini S, Rhind SG, Baker AJ, Shek PN, Tien H (2009). Abnormal coagulation tests are associated with progression of traumatic intracranial hemorrhage. J Trauma.

[33] Carnevale JA, Segar DJ, Powers AY, Shah M, Doberstein C, Drapcho B (2018). Blossoming contusions: identifying factors contributing to the expansion of traumatic intracerebral hemorrhage. J Neurosurg.

[34] Zeiler FA, Donnelly J, Nourallah B, Thelin EP, Calviello L, Smielewski P (2018). Intracranial and extracranial injury burden as drivers of impaired cerebrovascular reactivity in traumatic brain injury. J Neurotrauma.

[35] Zeiler FA, Ercole A, Cabeleira M, Carbonara M, Stochetti N, Menon DK, Smielewski P, Czosnyka M (2019). Comparison of performance of different optimal cerebral perfusion pressure parameters for outcome prediction in adult traumatic brain injury: A collaborative european neurotrauma effectiveness research in traumatic brain injury (CENTER-TBI) study. J Neurotrauma.

[36] Brady KM, Lee JK, Kibler KK, Easley RB, Koehler RC, Shaffner DH (2008). Continuous measurement of autoregulation by spontaneous fluctuations in cerebral perfusion pressure: comparison of 3 methods. Stroke.

[37] Hardman JM, Manoukian A (2002). Pathology of head trauma. Neuroimaging Clin N Am.

[38] Matsuyama T, Shimomura T, Okumura Y, Sakaki T (1997). Rapid resolution of symptomatic acute subdural hematoma: case report. Surg Neurol.

